# Ageism on Campus: Opportunities to Include Age in Diversity and Inclusion Efforts

**DOI:** 10.1093/geroni/igaa057.021

**Published:** 2020-12-16

**Authors:** Cassandra Barragan, Stephanie Wladkowski

**Affiliations:** Eastern Michigan University, Ypsilanti, Michigan, United States

## Abstract

Diversity and inclusion are essential perspectives on university campuses. In recent years, there has been a nationwide decline in admissions resulting in changes to traditionally FTIAC driven college campuses. An environmental scan was completed at a mid-sized midwestern university to explore age-inclusive barriers and opportunities for change. In-depth interviews were held with 28 EMU stakeholders representing a wide variety of ages in leadership positions across campus. Students aged 40 and above (N=248) were also surveyed about their experiences on campus. Qualitative analysis revealed ageist attitudes about older adults and older students from at all levels of the university. Results demonstrate that initial responses to ‘age-friendly’ focused on stereotypes of older adults, but attitudes adjusted when reframed as older learners and further refined when older learners were defined as 40 and above. Additionally, there was a distinct disconnect between ageist perceptions towards older adults and older students which highlights the importance of intergenerational opportunities as an approach to combat ageist attitudes on campus. While these barriers require long-term and complicated solutions, participants described the many benefits that older learners bring to enrich the campus. Results of this research revealed opportunities to reframe aging in the context of diversity and inclusion efforts on campus. Adopting diversity efforts to include age can benefit universities in not only admissions, classroom experiences, and connections to surrounding communities.

